# Sweet's syndrome in a patient with Crohn's disease: a case report

**DOI:** 10.1186/1752-1947-2-221

**Published:** 2008-06-28

**Authors:** Nadia M Mustafa, Mark Lavizzo

**Affiliations:** 1 Internal Medicine Residency Program, College of Medicine, University of Illinois at Urbana-Champaign, USA; 2 Assistant professor, College of Medicine, University of Illinois at Urbana-Champaign, USA

## Abstract

**Background:**

Sweet's syndrome, also known as acute febrile neutrophilic dermatosis, has been associated with malignancy, autoimmune disease and collagen vascular disease. The association of Crohn's disease and Sweet's syndrome is rare. We report a case of Sweet's syndrome in a patient with Crohn's disease.

**Case presentation:**

A 63-year-old man with a history of Crohn's disease presented with one-week duration of abdominal pain, diarrhea and hematochezia. He also noticed eruption of painful skin rashes all over his body at the same time. Colonoscopy and esophagogastroduodenoscopy (EGD) showed inflammation involving different parts of the gastrointestinal tract consistent with Crohn's disease. Punch biopsy of the skin lesion was consistent with Sweet's syndrome, which has a rare association with Crohn's disease.

**Conclusion:**

Crohn's disease should be excluded in patients presenting with Sweet's syndrome and diarrhea. Alternatively, Sweet's syndrome should be considered as a diagnosis when a patient with Crohn's disease develops skin lesions.

## Introduction

Sweet's syndrome, also known as acute febrile neutrophilic dermatosis, has rarely been associated with Crohn's disease. We report a case of Sweet's syndrome in a patient with Crohn's disease.

## Case Presentation

**Figure 1:**
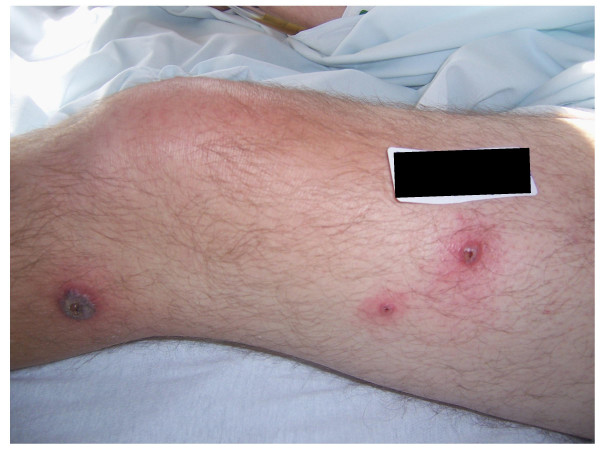
Pustular lesions with central necrosis on the patient's leg.

**Figure 2:**
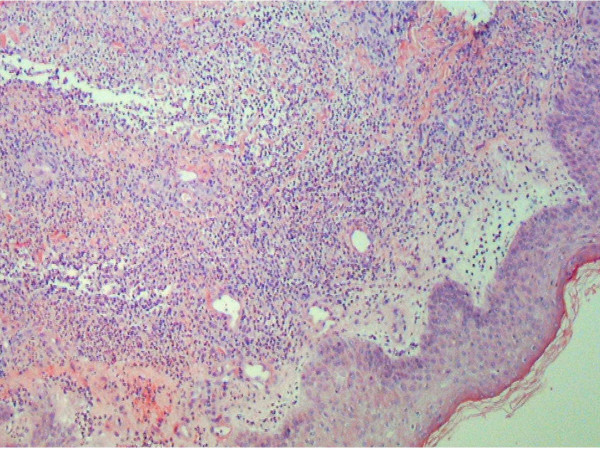
Punch biopsy of a skin lesion showing neutrophilic infiltration in the dermis, with no evidence of vasculitis.

A 63 year-old man with a history of Crohn's disease for the past thirty years and hyperlipidemia presented with one week of abdominal pain, diarrhea and hematochezia. Abdominal pain was generalized, 6 by 10 in intensity on the pain scale, and dull in character. It was worsened by food intake and relieved by bowel movement. The abdominal pain was associated with fever, chills, nausea and vomiting. The patient also complained of painful rashes all over his body that had erupted suddenly about a week ago. The rashes were nonpruritic and had started on the dorsum of his hands and spread to involve his face, neck, chest and legs. He denied using any new creams, soaps, detergents or perfumes or any change in his bed sheets or clothing. He also denied contact with pets, recent travel, a similar rash in any other family member, or being bitten by an insect. He denied having had any similar rash in the past. He had a history of Crohn's disease for the past thirty years which had been in remission for several years, until the past few months when he began to have episodes of diarrhea and rectal bleeding. Colonoscopy two years ago had showed inflammatory bowel disease of segmental nature with rectal sparing and primarily involving the ascending and sigmoid colon. His medications included Asacol which he had been taking for past few months and azathioprine which was started two weeks prior to his admission. He had previously been on prednisone which was started two months earlier with his last dose being four days prior to admission. His vital signs on presentation were: Temperature 100.5°F, blood pressure 95/58 mmHg, heart rate 120/min and respiratory rate 21 b/min. On physical examination his abdomen was mildly distended with tenderness to palpation in the left lower quadrant. He also had a papular rash and plaques, with surrounding erythema, scattered over his face, neck, chest and legs. (See Figure 1) These lesions were tender to palpation. Laboratory results showed an elevated white blood count (WBC) of 20.7 × 10^9 ^with 78% neutrophils and 14% bands. Comprehensive metabolic panel was significant for low sodium of 133 mEq/L and mildly elevated renal function with a blood urea nitrogen of 20 mg/dL and creatinine of 1.3 mg/dL and. His erythrocyte sedimentation rate (ESR) and C reactive protein were also high at 49 mm/hr and 161.6 mg/L respectively. Blood cultures were negative. Other laboratory tests, which included fungal serology, potassium hydroxide mount, gram stain, acid fast bacilli smear, bacterial culture, fungal culture and an acid fast culture of the skin rash, were all negative. He was started empirically on intravenous vancomycin for possible Methicillin Resistant *Staphylococcus Aureus folliculitis*, pending the results of investigations. Computed tomography (CT) scan of the abdomen on admission showed inflammation involving the colon and gastric and duodenal regions. Magnetic resonance angiography (MRA) of the abdomen was negative for mesenteric artery occlusion. Colonoscopy and esophagogastroduodenoscopy revealed pancolitis and gastroduodenitis consistent with Crohn's disease. Biopsy specimens taken from stomach, duodenum, ileum, ileocecal valve and colon revealed pancolitis, duodenitis and gastritis with no evidence of granuloma. The patient was diagnosed with an exacerbation of Crohn's disease and started on intravenous methylprednisolone 60 mg q 12 hrs, with continuation of azathioprine and Asacol. He was also given a dose of intravenous Infliximab. The rash showed no improvement after three days of antibiotics. A punch biopsy of one of the skin lesions revealed dense dermal infiltrate composed predominantly of neutrophils, with no evidence of vasculitis. This was consistent with the diagnosis of Sweet's syndrome. (Figure 2). Antibiotic treatment was stopped. The patient's symptoms and rash rapidly improved with systemic corticosteroid treatment.

## Discussion

Sweet's syndrome, also known as acute febrile neutrophilic dermatosis, was first described by Robert Douglas Sweet in 1964 [1]. Sweet's syndrome is characterized by fever, neutrophilia, cutaneous eruptions consisting of erythematous papules and plaques, and a dermal nonvasculitic neutrophilic infiltration on skin biopsy [2,3]. These plaques are painful but nonpruritic [4]. Other skin manifestations such as pustules, vesicles, purpura, ulcers and hemorrhagic lesions have been described [1]. Seventy-five percent of patients have some prodromal illness, most commonly an upper respiratory tract infection [5].

Common complications of Sweet's syndrome include arthralgia, arthritis, conjunctivitis, iridocyclitis, and rarely involvement of the central nervous system [4]. Sweet's syndrome is more common in females with a female to male ratio of 3.7:1, with the mean age of 52 years [1].

Sweet's syndrome should be regarded as a cutaneous marker of systemic disease. It has been associated with malignancies in about 20 to 25% of patients [6]. Most malignancies are hematopoietic, especially myelodysplastic syndromes and acute myeloid leukemia. Fifteen percent are due to solid tumors including breast, genitourinary and gastrointestinal malignancies [7]. Other causes of Sweet's syndrome are listed in table 1.

**Table 1:** Causes of Sweet's syndrome

Malignancies
Hematopeitic: myelodysplastic syndromes and acute myeloid leukemia, hairy cell leukemia, B and T cell lymphoma, agnogenic myeloid metaplasia
Solid tumors: breast, testicular, prostate, ovarian, vaginal squamous cell, genitourinary and gastrointestinal malignancies

Viral infections
Chronic active hepatitis, cytomegalovirus, human immunodeficiency virus

Bacterial infections
*Streptococcus, mycobacterium, yersinia, typhus, salmonella*

Autoimmune and collagen vascular diseases
Rheumatoid arthritis, systemic lupus erythematosus, mixed connective tissue disease, hashimoto thyroiditis, Sjogren's disease, behcet's disease

Medications
Furosemide, hydralazine, lithium, oral contraceptive pills, trimethoprim- sulfamethoxazole, minocycline and imatinib mesylate

Inflammatory bowel disease
Ulcerative Colitis, Crohn's disease

Pregnancy
Complement deficiency
Subacute necrotizing lymhadenitis
POEMS syndrome

Only a few cases of Sweet's syndrome associated with Crohn's disease have been reported in the literature [1]. There is a higher incidence of colonic involvement and extraintestinal features in these patients. The skin lesions have been observed in patients with active Crohn's disease, but sometimes it can precede the onset of intestinal symptoms. It appears that the syndrome is not initiated by the underlying disease but rather shares with it a concurrent pathogenic mechanism.

The pathogenesis of Sweet's syndrome is poorly understood. Cytokines, such as granulocyte colony stimulating factor (G-CSF), interleukin (IL)-1, IL-6, or IL-8, if deposited in the dermis, may be responsible for the immunopathologic and clinical manifestations of Sweet's syndrome. The fact that Sweet's syndrome can occur after G-

CSF treatment shows that IL-1, which is produced by acute myelocytic leukemia (AML) cells and stimulates the G-CSF gene, plays a role in the pathogenesis of Sweet's syndrome [1].

For a definitive diagnosis of Sweet's syndrome, both major and two minor criteria should be met. The two major criteria are 1) abrupt onset of painful erythematous plaques or nodules occasionally with vesicles, pustules, or bullae and, 2) neutophilic infiltration in the dermis without leukocytoclastic vasculitis. The minor criteria are 1) skin lesions preceded by a nonspecific respiratory or gastrointestinal tract infection, vaccination or associated with inflammatory diseases such as autoimmune disorders, infections, hemoproliferative disorders, solid malignant tumors or pregnancy, 2) accompanied by periods of general malaise and fever (> 38°C), 3) laboratory values during onset: ESR > 20 mm, C reactive protein positive, segmented neutrophils >70% in peripheral blood smear, leukocytosis > 8000 (3 of 4 of these values are necessary), and 4) excellent response to treatment with systemic corticosteroids or potassium iodide [1,8].

Sweet's syndrome is one of the groups of neutrophilic dermatoses that include pyoderma gangrenosum and whose association with ulcerative colitis and Crohn's disease is well established. Sweet's syndrome can be distinguished from pyoderma gangrenosum by the absence of vasculitis and lack of dermal necrosis, but histological features may occasionally overlap. The abrupt tendency for Sweet's syndrome to form multiple eruptions on the upper half of the body and the lack of ulceration also distinguishes the rash from pyoderma gangrenosum. However, the two conditions can occur in the same patient, as may other neutrophilic dermatosis, vesiculopapular eruptions or other cutaneous features of inflammatory bowel disease such as erythema nodosum or polyarthritis. The simultaneous occurrence of different rashes in the same person can be viewed as the dermatological expression of a neutrophilic reaction to a common stimulus [9].

Sweet's syndrome, if left untreated, usually heals within six to eight weeks [5].

Prednisone at an initial dose of 40–60 mg per day, with gradual tapering off over four to six weeks, is the standard treatment for Sweet's syndrome [3,5]. Relapses are common if steroids are tapered too quickly. In recurrent disease, therapy with colchicine, potassium iodide, dapsone, doxycycline, indomethacin, clofazimine, isotretinoin and cyclosporine have all been described [1,5].

Potassium iodide administered orally as 300 mg enteric-coated tablets, 3 times each day, for a daily dose of 900 mg, or as a saturated solution of potassium iodide (Lugol's solution), beginning at a dose of 3 drops 3 times each day (9 drops/day = 450 mg per day) and increasing by 1 drop 3 times per day, typically to a final dose of 21 drops/day (1050 mg) to 30 drops/day (1500 mg), usually results in resolution of fever and other symptoms within 1 to 2 days and resolution of skin lesions within 3 to 5 days of initiation of therapy. Vasculitis and hypothyroidism are potential adverse effects of potassium iodide [10].

## Conclusion

Sweet's syndrome should be considered an extraintestinal manifestation of Crohn's disease, and should be differentiated from other more frequent inflammatory diseases that accompany Crohn's disease, like erythema nodusum, pyoderma gangrenosum and leukocytoclastic vasculitis. Awareness of this association may guide appropriate diagnostic procedures and therapy.

## Competing interests

The authors declare that they have no competing interests.

## Authors' contributions

NM was involved in the management of the patient while in hospital, wrote the manuscript, collected all relevant data, and finalized the manuscript for submission to the journal, ML was involved in giving intellectual advice and reviewing the manuscript.

## Consent

Written informed consent was obtained from the patient for publication of this case report and any accompanying images.
